# Vaccination in India

**Published:** 1894-03-03

**Authors:** 


### VACCINATION IN INDIA.

Dr. Withington writes: With regard to the notice in The Hospital of February 24th, of a supposed ancient practice of vaccination in India, the following extract from De Haas' paper "Ueber die Ursprünge der Indischen Medicin" (Zeitschrift der Deutschen Morgenlandischen Gesellxhaft xxx., 661), may be of interest. "Besides nose-making, another discovery of which modern science is justly proud, has been claimed for the Hindus, that of vaccination. Ainslie ('Transactions of the Royal Asiatic Society' ii. 67) quotes from the *Madras Courier* of January 12th, 1819, two passages which a learned Hindu professed to have found in the Sâkteya Grantha, a work ascribed to Dhanwankari (the Hindu god of medicine). One is given in Sanskrit with an English translation, the other in English only. The Sanskrit of the first is obscure and irregular, and shows obvious signs of being an unskilful translation from a foreign source 'Take the fluid of cow-pox from a cow's udder, or a man's upper arm, on a lancet, wound another man's upper arm till the blood flows, then, when the matter mingles with the blood cow-pox will be produced.' The second (quoted in The Hospital) is clear enough, but also has a decidedly modern appearance, and certinly never existed in that form in Sanskrit. Ainslie says he is incompetent to decide the genuineness of the Sanskrit, but expresses well founded doubts on the matter, for the simple reason that cow-pox does not occur (naturally) in hot climates, and was with difficulty introduced into India in 1802." Dr. Haas thinks there is little doubt that the "learned Hindu" inserted these passages himself, possibly at the instgiation of English doctors or officials, in order to overcome the native prejudices against vaccination. I have not seen any report of Dr. Pringle's lecture, but if he has no further evidence than the above his conclnsion seems scarcely warranted.

